# Integration of Gene Expression Profile Data to Screen and Verify Hub Genes Involved in Osteoarthritis

**DOI:** 10.1155/2018/9482726

**Published:** 2018-08-14

**Authors:** Zhaoyan Li, Qingyu Wang, Gaoyang Chen, Xin Li, Qiwei Yang, Zhenwu Du, Ming Ren, Yang Song, Guizhen Zhang

**Affiliations:** ^1^Department of Orthopedics of the Second Hospital of Jilin University, Ziqiang Street 218, Changchun, Jilin 130041, China; ^2^Research Centre of the Second Hospital of Jilin University, Ziqiang Street 218, Changchun, Jilin 130041, China; ^3^The Engineering Research Centre of Molecular Diagnosis and Cell Treatment for Metabolic Bone Diseases of Jilin Province, Ziqiang Street 218, Changchun, Jilin 130041, China

## Abstract

Osteoarthritis (OA) is one of the most common diseases worldwide, but the pathogenic genes and pathways are largely unclear. The aim of this study was to screen and verify hub genes involved in OA and explore potential molecular mechanisms. The expression profiles of GSE12021 and GSE55235 were downloaded from the Gene Expression Omnibus (GEO) database, which contained 39 samples, including 20 osteoarthritis synovial membranes and 19 matched normal synovial membranes. The raw data were integrated to obtain differentially expressed genes (DEGs) and were deeply analyzed by bioinformatics methods. The Gene Ontology (GO) and pathway enrichment of DEGs were performed by DAVID and Kyoto Encyclopedia of Genes and Genomes (KEGG) online analyses, respectively. The protein-protein interaction (PPI) networks of the DEGs were constructed based on data from the STRING database. The top 10 hub genes VEGFA, IL6, JUN, IL1*β*, MYC, IL4, PTGS2, ATF3, EGR1, and DUSP1 were identified from the PPI network. Module analysis revealed that OA was associated with significant pathways including TNF signaling pathway, cytokine-cytokine receptor interaction, and osteoclast differentiation. The qRT-PCR result showed that the expression level of IL6, VEGFA, JUN, IL-1*β*, and ATF3 was significantly increased in OA samples (p < 0.05), and these candidate genes could be used as potential diagnostic biomarkers and therapeutic targets of OA.

## 1. Introduction

Osteoarthritis is a chronic joint disease characterized by degeneration of cartilage, synovial inflammation osteophytes formation, and subchondral bone sclerosis. Its typical signs and symptoms include pain, swelling, and stiffness, often accompanied by a decrease in function and limitation of movement [1]. It is a slowly progressive, disabling joint disorder that significantly reduces the quality of life. By 2030, it is predicted that 67 million people in the United States will be diagnosed with OA [2]. Although there are extensive studies on the mechanism and etiology in OA formation and progression, the causes of OA are still not clear.

Epidemiological studies have demonstrated that OA is a complex polygenic disorder with numerous environmental and genetic risk factors, in which one of the contributing factors to disease progression is a genetic component [3]. Over the last 15 years, researches have focused on the search for susceptible sites of osteoarthritis. Genomewide association studies (GWAS) could discover potential genetic variants that could be used as biomarkers for early diagnosis and targeted therapy [4]. At present, with the development of high-throughput sequencing technology, a large number of studies have been performed on osteoarthritis gene expression profiles and screened thousands of differentially expressed genes. However, the results for the expressed mRNAs are inconsistent with different gene profile due to sample heterogeneity or different sequencing platform. Thus, no reliable results have been identified in OA. However, the integrated bioinformatics methods will solve the disadvantages and identify the hub genes involved in OA.

In this work, we have downloaded two microarray datasets GSE12021 [5] and GSE55235 [6] and screened out differentially expressed genes (DEGs) between synovial membranes of knee OA patients and normal controls. GO and pathways enrichment analyses of DEGs were applied and functional module analysis of the protein-protein interaction (PPI) network was also constructed. The study aimed to identify hub genes and explore the intrinsic molecular mechanisms involved in OA.

## 2. Materials and Methods

### 2.1. Microarray Data Information

The Gene Expression Omnibus (GEO, http://www.ncbi.nlm.nih.gov/geo) is a public genomics data repository which stores gene expression datasets and original series and platform records [7]. The gene expression profiles of GSE12021 and GSE55235 were downloaded from the GEO database which all based on GPL96 (Affymetrix Human Genome U133A Array) platform. The microarray data of GSE12021 include 10 knee osteoarthritis synovial membranes and 9 normal controls, and the microarray data of GSE55235 include 10 knee osteoarthritis synovial membranes and 10 normal controls.

### 2.2. Data Processing and Identification of DEGs

The process of data preprocessing included background adjustment, normalization, and summarization. The raw data were preprocessed by affy package [8] in R software and limma package [9] in R software was used to identify the upregulated and downregulated DEGs between osteoarthritis synovial membranes and normal controls. P values were adjusted using the Benjamini and Hochberg test, and p < 0.05 and |log⁡FC| > 1 were considered as the cutoff criterion.

### 2.3. Gene Ontology (GO) and Pathway Enrichment Analyses

DAVID (the Database for Annotation, Visualization, and Integrated Discovery) online bioinformatics database integrates biological data and analysis tools to provide systematic annotation information for biological function of large-scale gene or protein list [10]. In the present study, Gene Ontology enrichment and KEGG (Kyoto Encyclopedia of Genes and Genomes) pathway analysis of DEGs were conducted using the DAVID online tool. GO analysis included categories of biological processes (BP), cellular component (CC), and molecular function (MF). Pathway analysis is a functional analysis that maps genes to KEGG pathways. And gene count >2 and p < 0.05 were set as the cutoff point.

### 2.4. Integration of Protein-Protein Interaction (PPI) Network Analysis

STRING (https://string-db.org/cgi/input.pl) is an online database resource search tool for the retrieval of interacting genes, which include physical and functional associations [11]. In this paper, the STRING online tool was used to construct a protein-protein interaction (PPI) network of upregulation and downregulation DEGs, with a confidence score >0.4 defined as significant. Then we imported the interaction data into the Cytoscape software [12] to map a PPI network. Based on the above data, we used Molecular Complex Detection (MCODE) [13], a built-in APP in Cytoscape software, to analyze the interaction relationship of the DEGs encoding proteins and screening hub gene. The parameters of network scoring and cluster finding were set as follows: degree cutoff = 2, node score cutoff = 0.2, k-core = 2, and max depth = 100.

### 2.5. qRT-PCR Validation and Statistical Analysis

Quantitative reverse transcription-PCR was used to validate the hub genes. Total RNA was reverse-transcribed to cDNA using PrimeScript RT reagent Kit with gDNA Eraser (TaKaRa, Japan) according to the manufacturer's instructions. Primer 5.0 software (PREMIER Biosoft, Palo Alto, CA, USA) was used to design primers, and a QuantStudio™ 7 Flex real-time PCR system (Applied Biosystems, Carlsbad, CA, USA) was used. Primers for mRNA are listed in Table 1. All samples were normalized to GAPDH. And the relative expression levels of each gene were calculated using 2−ΔΔCt methods. Statistical analysis was performed with SPSS software (version 18.0 SPSS Inc.). P values < 0.05 were considered statistically significant.

### 2.6. Patients and Controls

Our study was approved by the ethics committee of the Second Hospital of Jilin University, Jilin University, Jilin, China. 10 healthy donors and 10 knee osteoarthritis patients with knee osteoarthritis (diagnosed according to the ACR classification criteria for knee osteoarthritis) [14] were enrolled, and all gave informed consent. Osteoarthritis synovial membrane samples were obtained from OA patients upon total knee replacement at the Second Hospital of Jilin University, and normal synovial membrane samples were obtained from traumatic joint injury cases upon joint synovectomy at the Second Hospital of Jilin University.

## 3. Results

### 3.1. Identification of DEGs in Osteoarthritis

A total of 20 osteoarthritis synovial membranes and 19 matched normal synovial membranes were analyzed; taking p < 0.05 and |log⁡FC| > 1 as a threshold, we extracted 1834 and 1948 DEGs from the expression profile datasets GSE12021 and GSE55235, respectively. By integrated analysis, a total of 258 DEGs were identified, including 161 upregulated DEGs and 97 downregulated DEGs in osteoarthritis samples compared with normal samples.

### 3.2. GO Functional Enrichment Analysis

**Figure 1:**
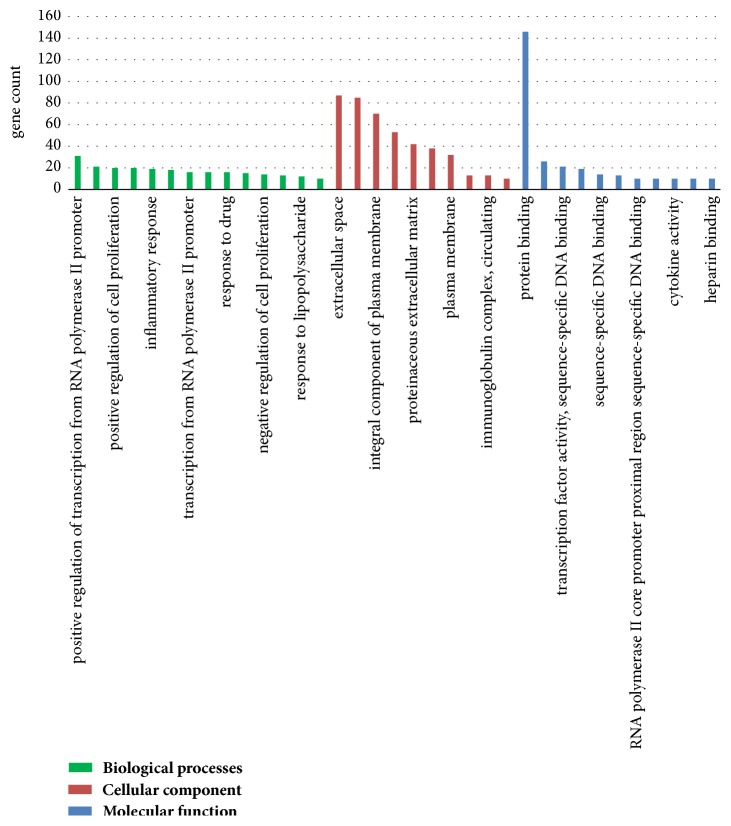
Gene Ontology analysis classified the DEGs into 3 groups: molecular function, biological process, and cellular component.

To acquire the functions of differential genes, GO function enrichment was analyzed by DAVID online tool, and the DEGs functions were classified into three groups as follows: BP, CC, and MF (Figure 1). As shown in the Figure 1 and Table 2, in the biological processes group, the down-DEGs are mainly enriched in phagocytosis, engulfment, innate immune response, positive regulation of MAP kinase activity, proteolysis, and complement activation, and the up-DEGs are mainly enriched in cellular response to fibroblast growth factor stimulus, response to cAMP, and negative regulation of apoptotic process. In the cellular component group, the down-DEGs are mainly enriched in extracellular space, extracellular region, immunoglobulin complex, and circulating, and the up-DEGs are mainly enriched in nucleus, nucleoplasm, cytoplasm, and cytosol. And in the molecular function group, the down-DEGs are mainly enriched in drug binding, immunoglobulin receptor binding, and growth factor activity, and the up-DEGs are mainly enriched in transcriptional activator activity, protein binding, poly(A) RNA binding, and MAP kinase tyrosine/serine/threonine phosphatase activity.

### 3.3. Signaling Pathway Analysis

**Figure 2:**
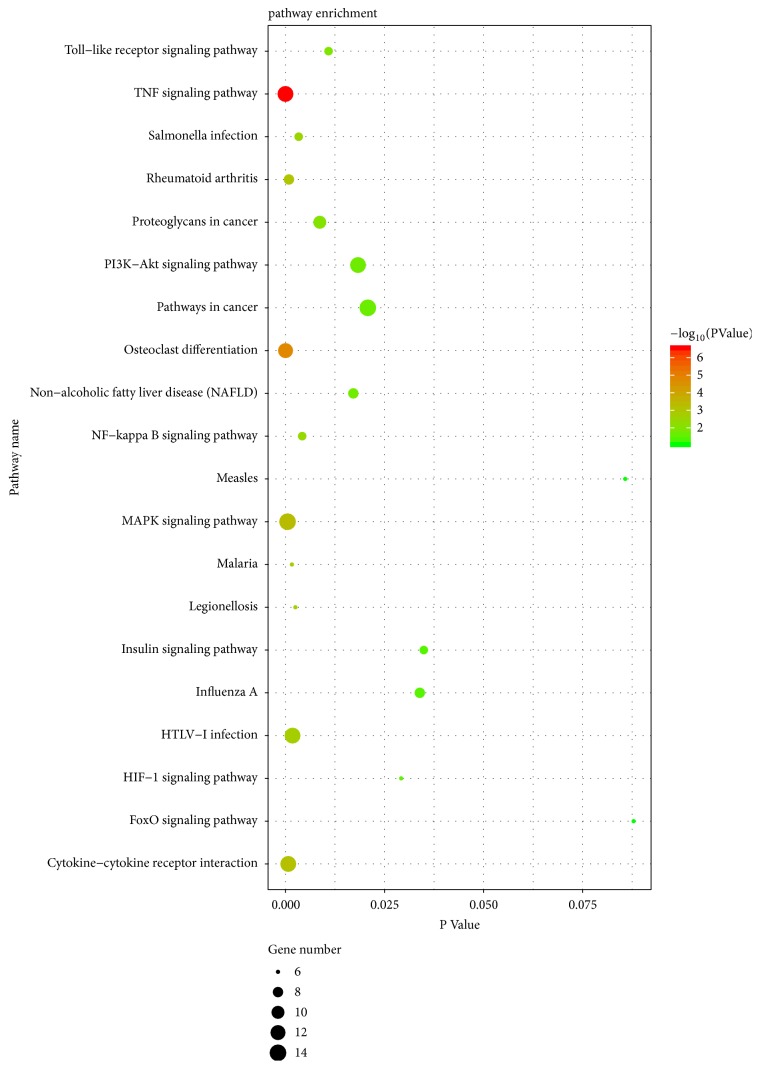
Kyoto Encyclopedia of Genes and Genomes (KEGG) enrichment analysis of the pathways. The gradual color represents the P value; the size of the black spots represents the gene number.

After the pathway enrichment analysis, downregulated genes were mainly enriched in cytokine-cytokine receptor interaction and glycosphingolipid biosynthesis-globoseries. And upregulated genes were mainly enriched in TNF signaling pathway, osteoclast differentiation, MAPK signaling pathway, NF-kappa B signaling pathway, and rheumatoid arthritis (Figure 2, Table 3).

### 3.4. PPI Network and Modular Analysis

**Figure 3:**
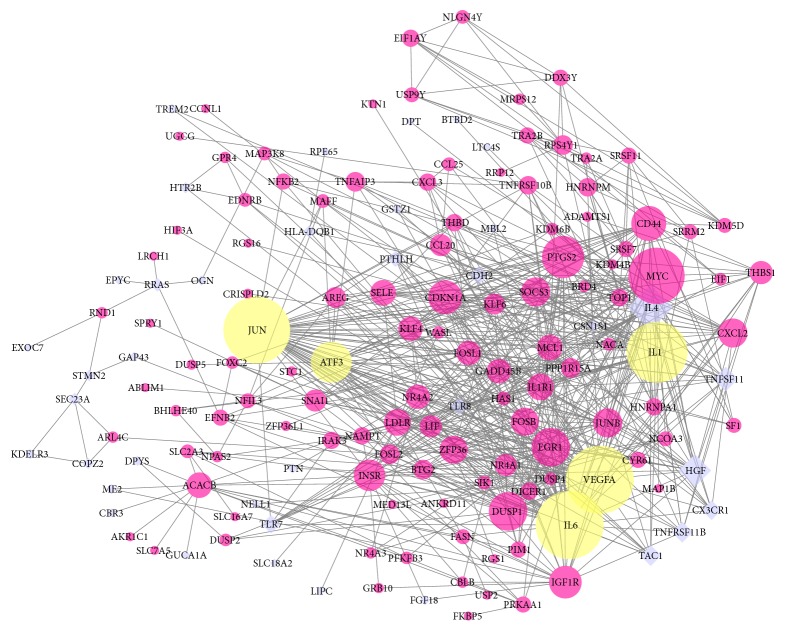
PPI network constructed with the upregulated and downregulated DEGs. Red nodes represent upregulated genes, purple nodes represent downregulated genes, and yellow nodes represent upregulated genes validated by qRT-PCR.

**Figure 4:**
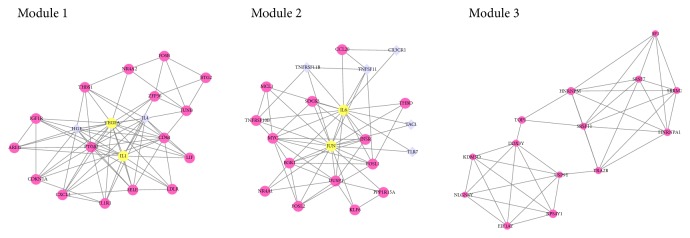
The three most significant modules. Red nodes represent upregulated genes, purple nodes represent downregulated genes, and yellow nodes represent upregulated genes validated by qRT-PCR.

Based on the data in the STRING database, we constructed a PPI network through Cytoscape software, containing 155 nodes and 625 edges (Figure 3). Among the 155 genes, the top 10 hub genes were identified according to connectivity, including VEGFA, IL6, JUN, IL-1*β*, MYC, IL4, PTGS2, ATF3, EGR1, and DUSP1. IL6 and VEGFA showed the highest degree (degree = 51). In order to further analyze the interaction of protein, 5 modules were detected using the Cytoscape plugin MCODE; the top thee module with score >5 were shown in Figure 4. In addition, functional enrichment analyses for these modules were performed. Pathway enrichment analysis showed that Module 1 is mainly associated with TNF signaling pathway, cytokine-cytokine receptor interaction, and osteoclast differentiation. Module 2 is mainly associated with osteoclast differentiation, TNF signaling pathway, and cytokine-cytokine receptor interaction. Module 3 is mainly associated with Spliceosome.

### 3.5. Validation of Hub Gene

**Figure 5:**
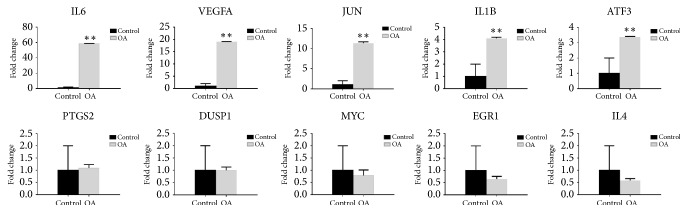
Validation of the top 10 hub genes by qRT-PCR between the OA group (n = 10) and the control group (n = 10). All samples were normalized to the expression of GAPDH, and the relative expression levels of each gene were analyzed using the 2−ΔΔCt method. ^*∗∗*^P < 0.01.

To validate microarray results, the expression levels of top 10 hub genes were determined in synovial membrane samples of knee osteoarthritis and normal controls using qRT-PCR. The verification result showed that the expression levels of IL6, VEGFA, JUN, IL-1*β*, and ATF3 were significantly increased in osteoarthritis samples (p < 0.05) (Figure 5). All validations are consistent with the analytical results in this study.

## 4. Discussion

OA is the most common degenerative joint disease observed worldwide. The prevalence of clinical osteoarthritis has grown to nearly 27 million in the USA [15], and it is a great burden on people's health and medical insurance; therefore, early diagnosis and treatment of osteoarthritis are especially important. Epidemiological studies have demonstrated that osteoarthritis is a multifactorial polygenic disease with numerous environmental and genetic risk factors [16]. It is important to study the molecular mechanisms of the OA. The previous study on the pathophysiology of osteoarthritis has focused on cartilage and periarticular bone and neglecting the role of synovial tissue in the pathogenesis of osteoarthritis. During OA progression, the synovial membrane is also a source of proinflammatory and catabolic products, and there are multiple pathways and mediators that can directly influence the development and persistence of synovitis [17].

Microarray and high-throughput sequencing technologies have been widely used to predict potential targets gene for osteoarthritis, but most studies focus on a single cohort study or single genetic event. This study integrated two cohorts profile datasets from different groups, and both samples are synovial membranes isolated from knee osteoarthritis patients. Bioinformatics methods are applied to analyze the raw data, and we identify 258 DEGs, including 161 upregulated DEGs and 97 downregulated DEGs. Next, the 258 EDGs were classified into three groups by GO terms using multiple approaches and further clustered based on functions and signaling pathways, respectively.

The DEGs in osteoarthritis analyzed by GO functional enrichment analysis showed that the downregulated DEGs were mainly enriched in immune response, proteolysis, positive regulation of MAP kinase activity, and growth factor activity, while upregulated DEGs were shown to be concerned with cellular response to fibroblast growth factor stimulus, response to cAMP, negative regulation of apoptotic process, and MAP kinase tyrosine. This conforms to our knowledge that immune response, inflammatory responses, and response to cAMP are main mechanisms of OA development and progression [18–22]. According to the previous studies, the participation of the immune system in the development and progression of OA is one of the key elements in the pathogenesis of the disease [23]. It should be noted that the pathophysiological processes occurring in OA are largely mediated by inflammatory cytokines and other anti-inflammatory cytokines that may modulate an inflammatory response and act protectively on joint tissue [24]. The main representatives of anti-inflammatory cytokines involved in the pathogenesis of OA are IL-4, IL-10, and IL-13. In our study, the downregulated gene IL-4 is enriched in immune response, and the dysregulated IL-4 may be involved in the pathogenesis of OA. Furthermore, the enriched KEGG pathways of DEGs and modules analysis included the TNF signaling pathway, MAPK signaling pathway, osteoclast differentiation, and cytokine-cytokine receptor interaction. Previous studies showed that these pathways are involved in osteoarthritis cartilage degeneration and synovial hyperplasia [25–28]. The PPI network was constructed with DEGs, and the top 10 hub genes were as follows: VEGFA, IL6, JUN, IL-1*β*, MYC, IL4, PTGS2, ATF3, EGR1, and DUSP1. The results validated by qRT-PCR show that the expression levels of IL6, VEGFA, JUN, IL-1*β*, and ATF3 were significantly increased in OA samples (p < 0.05).

Module analysis of the PPI network suggested that TNF signaling pathway, cytokine-cytokine receptor interaction, and osteoclast differentiation might be involved in OA development. Tumor necrosis factor (TNF) is a critical cytokine, which can induce a wide range of intracellular signal pathways including apoptosis and cell survival as well as inflammation and immunity [29]. TNF-*α* mediated activation of NF-*κ*B signaling pathway is known to play an important role in the pathogenesis of OA [30], and OA was effectively treated by VIP via inhibiting the NF-*κ*B signaling pathway [31]. Cytokines which are produced in joint tissues regulate a broad range of inflammatory processes [32]; the occurrence and development of OA are driven by various mediators, of which the key role is attributed to the interactions within the cytokine-cytokine network. The inflammatory and anti-inflammatory cytokines play a key role in the pathogenesis of OA [33]. Proinflammatory cytokines such as TNF-a, IL-1 *β*, and IL-17 can enhance osteoclast formation [34]. The role of osteoclasts in chronic arthritis has emerged in recent years; osteoclasts may be key players in the erosive and inflammatory events leading to joint destruction and bone resorption [35].

According to the recent report, the immune system is one of the key elements in the pathogenesis of the OA [23]. Inflammatory cytokines, including IL-1*β*, TNF*α*, IL-6, and IL-18, play an important role in the development and progression of disease [24]. In our study, inflammatory cytokines IL6 and IL-1*β* were significantly increased in OA samples. IL-1*β* is one of the representatives of the IL-1 family; the protein encoding by IL-1*β* induces inflammatory reactions and catabolic effect. The level of IL-1*β* has elevated in the synovial membrane, synovial fluid, cartilage, and the subchondral bone [36, 37]. The biological activation of synovial cells by IL-1*β* is mediated by the interaction between IL-1R1 and IL-1R2 receptor; blocking their connection with IL-1*β* may decrease in the activity of IL-1*β* [38]. Downregulation of the production and activity of active proinflammatory and procatabolic IL-1*β* is optimal for OA molecular therapy [39]. IL6 gene encodes a cytokine that strongly activates the immune system and inflammatory response; the protein is primarily produced at sites of acute and chronic inflammation, where it is secreted into the serum and induces a transcriptional inflammatory response through interleukin 6 receptor, alpha. The production of IL-6 in the degenerative joint is usually in response to IL-1*β* and TNF*α* and is mainly implemented by chondrocytes and osteoblasts [40], cooperating with IL-1*β* and TNF*α*, activation of osteoclasts formation, and thus bone resorption [41]. In synergy with another cytokine, IL6 causes an increase in the production of enzymes and decrease in type II collagen [42].

VEGFA is the founding member of the VEGF family and is the most widely studied gene in the molecular mechanism of OA. VEGFA gene encodes a heparin-binding protein, which induces proliferation and migration of vascular endothelial cells, and is essential for both physiological and pathological angiogenesis. VEGF is an important mediator of bone development [43]. Increased VEGF levels are associated with OA progression, and it is involved in pathologies including synovitis, cartilage degeneration, osteophyte formation, and pain [44]. During the advanced stage of OA, VEGF expression has been found increased in the articular cartilage and synovium [45]. Synergy with IL-1*β*, VEGF was found to significantly reduce the expression of aggrecan and type II collagen at the gene and protein levels [46]. VEGF expression increased in synovial macrophages and fibroblast-like synovial cells, and the expression of TNF-*α* and IL-6 [47] increased as well. There is growing evidence suggesting the pathological involvement of VEGF and its signaling pathways. Treatments targeting VEGF signaling will be a supplement of traditional treatments in OA.

The involvement of c-Jun N-terminal kinase (JNK) in signaling transduction pathways has been well-characterized in articular chondrocytes [48]. The basic leucine zipper transcription factor, ATF-like (BATF), a member of the Activator protein-1 family (AP-1), promotes transcriptional activation or repression, depending on the interacting partners (JUN-B or C-JUN), BATF, which forms a heterodimeric complex with JUN-B, and C-JUN may play important roles in OA cartilage destruction through regulating anabolic and catabolic gene expression in chondrocytes [49]. The involvement of ATF3 in joint disease has not been well studied, the ATF3 gene which belongs to the ATF/cAMP-responsive element-binding protein family and encodes a member of the activating transcription factor [50]. ATF3 expression significantly increased in the OA cartilage, and ATF3 deficiency decreased cytokine-induced IL6 transcription in chondrocytes through repressing NF-kB signaling. The deficiency of ATF3 may alleviate articulfvar degeneration of OA patient; ATF3 and its related pathways may be a suitable drug target for the treatment of OA [51, 52].

In summary, by means of data processing and qRT-PCR validation, the hub genes including IL6, VEGFA, JUN, IL-1*β*, and ATF3 may have the potential to be used as drug targets and diagnostic markers of OA. Although several hub genes and pathways were identified and validated in our study, there were still some limitations: small sample size was used for the analyses and there was lack of further experiment. Further experimental studies with larger sample size are needed to confirm our analysis result.

## Figures and Tables

**Table 1:** The primers of top 10 hub genes.

**Gene name**	**Forward primer**	**Reverse primer**
VEGFA	GTGAATGCAGACCAAAGAAAGA	AGGCTCCAGGGCATTAGAC
IL6	TCAATATTAGAGTCTCAACCCCCA	GAAGGCGCTTGTGGAGAAGG
JUN	GAGCTGGAGCGCCTGATAAT	CCCTCCTGCTCATCTGTCAC
IL1B	TGAGCTCGCCAGTGAAATGA	AGGAGCACTTCATCTGTTTAGGG
MYC	TACAACACCCGAGCAAGGAC	GAGGCTGCTGGTTTTCCACT
IL4	CATCTTTGCTGCCTCCAAGAACA	GTTCCTGTCGAGCCGTTTCA
PTGS2	GCTGTTCCCACCCATGTCAA	AAATTCCGGTGTTGAGCAGT
ATF3	GAGGTGGGGTTAGCTTCAGT	TCATTTTGATTTTGGGGCAAGGT
EGR1	CACCTGACCGCAGAGTCTTT	GAGTGGTTTGGCTGGGGTAA
DUSP1	CTCAAAGGAGGATACGAAGCGTT	CCCTGATCGTAGAGTGGGGT

**Table 2:** The significant enriched analysis of DEGs in osteoarthritis.

**Expression**	**Category**	**Term**	**Description**	**Gene Count**	**P-Value**
DOWN-DEGs	BP	GO:0006911	phagocytosis, engulfment	5	3.11E-05
	BP	GO:0045087	innate immune response	9	0.001731552
	BP	GO:0043406	positive regulation of MAP kinase activity	4	0.003542436
	BP	GO:0006508	proteolysis	9	0.004361459
	BP	GO:0006956	complement activation	4	0.010412249
	CC	GO:0005615	extracellular space	24	8.12E-08
	CC	GO:0005576	extracellular region	23	6.94E-06
	CC	GO:0042571	immunoglobulin complex, circulating	3	0.003820636
	CC	GO:0005886	plasma membrane	32	0.005732121
	CC	GO:0000139	Golgi membrane	9	0.008249883
	MF	GO:0004252	serine-type endopeptidase activity	7	0.001703259
	MF	GO:0008144	drug binding	4	0.006434471
	MF	GO:0034987	immunoglobulin receptor binding	3	0.007358675
	MF	GO:0008201	heparin binding	5	0.008337436
	MF	GO:0008083	growth factor activity	5	0.008701217

UP-DEGs	BP	GO:0044344	cellular response to fibroblast growth factor stimulus	7	2.20E-07
	BP	GO:0045944	positive regulation of transcription from RNA polymerase II promoter	27	4.77E-07
	BP	GO:0051591	response to cAMP	7	3.08E-06
	BP	GO:0000122	negative regulation of transcription from RNA polymerase II promoter	21	5.86E-06
	BP	GO:0043066	negative regulation of apoptotic process	16	1.31E-05
	CC	GO:0005634	nucleus	75	6.25E-07
	CC	GO:0005654	nucleoplasm	47	2.11E-06
	CC	GO:0005737	cytoplasm	61	0.002569078
	CC	GO:0005829	cytosol	43	0.002630227
	CC	GO:0005667	transcription factor complex	6	0.02296627
	MF	GO:0001077	transcriptional activator activity, RNA polymerase II core promoter proximal region sequence-specific binding	12	1.03E-05
	MF	GO:0005515	protein binding	106	2.10E-05
	MF	GO:0000982	transcription factor activity, RNA polymerase II core promoter proximal region sequence-specific binding	5	5.02E-05
	MF	GO:0044822	poly(A) RNA binding	25	7.91E-05
	MF	GO:0017017	MAP kinase tyrosine/serine/threonine phosphatase activity	4	1.95E-04

**Table 3:** Signaling pathway enrichment analysis of DEGs function in osteoarthritis.

**Expression**	**Term**	**Description**	**Gene Count**	**P-Value**
DOWN- DEGs	hsa04060	Cytokine-cytokine receptor interaction	5	0.036430433
	hsa00603	Glycosphingolipid biosynthesis - globoseries	2	0.074364973

UP-DEGs	hsa04668	TNF signaling pathway	13	3.53E-09
	hsa04380	Osteoclast differentiation	9	1.61E-04
	hsa04010	MAPK signaling pathway	12	2.13E-04
	hsa05134	Legionellosis	6	4.41E-04
	hsa05132	Salmonella infection	7	4.52E-04
	hsa05219	Bladder cancer	5	0.001397716
	hsa05144	Malaria	5	0.002719238
	hsa05166	HTLV-I infection	10	0.003418411
	hsa04064	NF-kappa B signaling pathway	6	0.003787905
	hsa05323	Rheumatoid arthritis	6	0.003978832

## Data Availability

The data used to support the findings of this study are available from the corresponding author upon request.

## Abbreviations

OA: Osteoarthritis
DEGs: Differentially expressed genes
GEO: Gene Expression Omnibus
PPI: Protein-protein interaction
GO: Gene Ontology
KEGG: Kyoto Encyclopedia of Genes and Genomes
GWAS: Genomewide association studies
BP: Biological processes
CC: Cellular component
MF: Molecular function
MCODE: Molecular Complex Detection
VEGFA: Vascular endothelial growth factor A
IL6: Interleukin 6
JUN: Jun protooncogene
IL1 *β*: Interleukin 1 beta
MYC: MYC protooncogene
IL4: Interleukin 4
PTGS2: Prostaglandin-endoperoxide synthase 2
ATF3: Activating transcription factor 3
EGR1: Early growth response 1
DUSP1: Dual specificity phosphatase 1
cAMP: Cyclic adenosine monophosphate
cDNA: DNA complementary to RNA
MAPK: Mitogen-activated protein kinase.
